# Low Carbohydrate Antigen 19-9 (CA 19-9) Levels in a Patient Highly Suspected of Having Caput Pancreas Tumor

**DOI:** 10.7759/cureus.24357

**Published:** 2022-04-21

**Authors:** Bernard Jonathan Christian Yong, Made Wirama Diyana

**Affiliations:** 1 Internal Medicine, Wangaya Regional Hospital, Bali, IDN

**Keywords:** pancreatic malignancy, serum tumor markers, ca 19-9, tumor marker, pancreatic cancer

## Abstract

Pancreatic cancer is the seventh leading cause of cancer-related mortality worldwide and the eleventh most common cause of cancer-related death in Indonesia. In pancreatic cancer, rapid and early diagnosis is crucial. Carbohydrate antigen 19-9 (CA 19-9), the most sensitive and specific tumor marker for pancreatic cancer, may help in diagnosing and determining prognosis; however, some populations do not express CA 19-9. Cases of low CA 19-9 may occur in populations with Lewis^⍺-β- ^genotype. These populations are not able to express Lewis antigen and CA 19-9; therefore, CA 19-9 investigation cannot be used for diagnostic and therapeutic measures. In patients highly suspicious of pancreatic cancer where CA 19-9 levels are low, alternative tumor markers such as CA 125 and carcinoembryonic antigen or a combination of various tumor markers can be used to increase sensitivity and specificity in diagnosing pancreatic cancer. A 70-year-old man presented with a complaint of worsening abdominal pain for the last two days. The patient had dark-yellow urine and pale stool. Abdominal ultrasonography and computed tomography scan showed a mass on the head of the pancreas, which was highly suspicious of pancreatic cancer.

## Introduction

The pancreas is an organ that functions as an exocrine and endocrine gland. In 2020, 5,781 cases of pancreatic cancer were reported. Pancreatic cancer is the seventh leading cause of cancer-related mortality globally [1]. There are various types of cancers based on the involved cells. Pancreatic cancer is typically classified into two types: pancreatic adenocarcinoma (85%), arising from the pancreatic exocrine gland, and pancreatic neuroendocrine tumor (Pan-NET) (5%), arising from the pancreatic endocrine gland [2]. In the early course of the disease, pancreatic cancer is usually asymptomatic; therefore, patients are usually diagnosed in the advanced stage with a poor prognosis and a survival rate of 1-3% in stage III-IV pancreatic adenocarcinoma [3].

The etiology of pancreatic cancer is multifactorial. Non-modifiable risk factors include age over 55 years, especially individuals 70-80 years old; male gender; blood types A, B, and AB; genetics; family history of pancreatic cancer; and type 2 diabetes, especially when the duration is over 10 years. Modifiable risk factors include gastrointestinal and intrapancreatic microflora; smoking or tobacco consumption; occupational exposure to metals or pesticides; alcohol consumption; high red-meat diet, processed meats, cholesterols, and nitrosamine; chronic pancreatitis; acute recurrent pancreatitis; and obesity [3,4].

Early-stage pancreatic cancer is usually asymptomatic. The non-specific symptoms gradually appear, including abdominal pain, jaundice, pruritus, dark-yellow urine, and pale stool due to biliary system obstruction. In addition, anorexia, significant weight loss, early satiety, dyspepsia, nausea, and malabsorption with steatorrhea may also present [3]. Although rapid diagnosis is crucial, pancreatic cancer screening in asymptomatic individuals without risk factors is not recommended [5]. Abdominal ultrasonography (USG) may be performed to evaluate pancreatic masses; however, computed tomography (CT) and magnetic resonance imaging (MRI) scans are the better imaging techniques. The gold standard for diagnosis is histopathological examination by biopsy of post-resected tumor [3].

Tumor markers that can help confirm the diagnosis and establish prognosis include carbohydrate antigen 19-9 (CA 19-9), carcinoembryonic antigen (CEA), and CA 125 [2]. CA 19-9 is the most sensitive (79%) and specific (82%) tumor marker for pancreatic cancer compared to other tumor markers; however, 10% of the population with negative Lewis genotype do not express CA 19-9 [3]. Furthermore, there are subgroups of pancreatic tumors with CA 19-9 levels that are not elevated or fluctuating; therefore, CA 19-9 cannot be used for diagnosis or post-treatment monitoring. In this case report, we present a case of a pancreatic tumor without increased CA 19-9 level.

## Case presentation

A 70-year-old man presented to the Emergency Room (ER) of Wangaya Regional Hospital with a complaint of worsening abdominal pain for the last two days which was mainly localized to the left upper quadrant of the abdomen, with a Visual Analog Scale (VAS) score of 4-5 which worsened after eating. The pain was persistent with nausea, vomiting, and weight loss. In the last year, the patient had experienced several episodes of similar abdominal pain, as well as fatigue and jaundice. Moreover, his urine became dark-yellow similar to tea, and, occasionally, he experienced pale watery stools; his weight decreased by about 10-15 kg. He denied any hematemesis and dark-colored stools. Physical examination showed anemic conjunctiva, icteric sclera, and a palpable mass on the epigastric region of the abdomen with tenderness and normal bowel movement. Before he came to Wangaya Regional Hospital, he admitted that he had sought several medical advice at a local public health center, and was diagnosed with dyspepsia.

**Figure 1:**
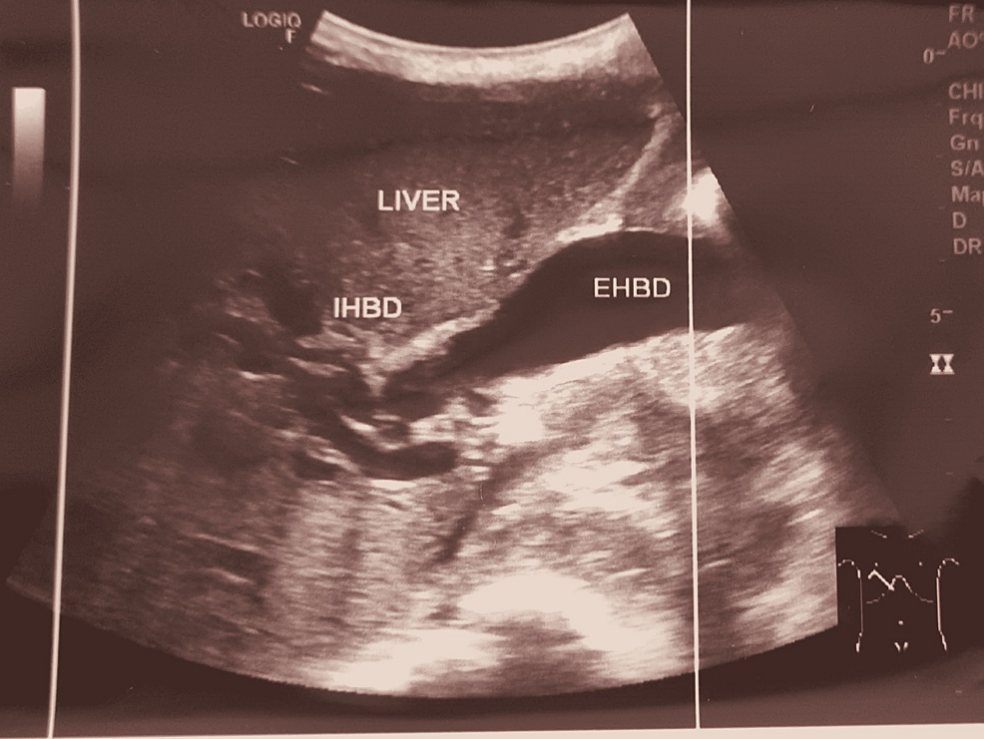
Dilated IHBD and EHBD on abdominal USG. IHBD: intrahepatic biliary duct; EHBD: extrahepatic biliary duct; USG: ultrasonography

**Figure 2:**
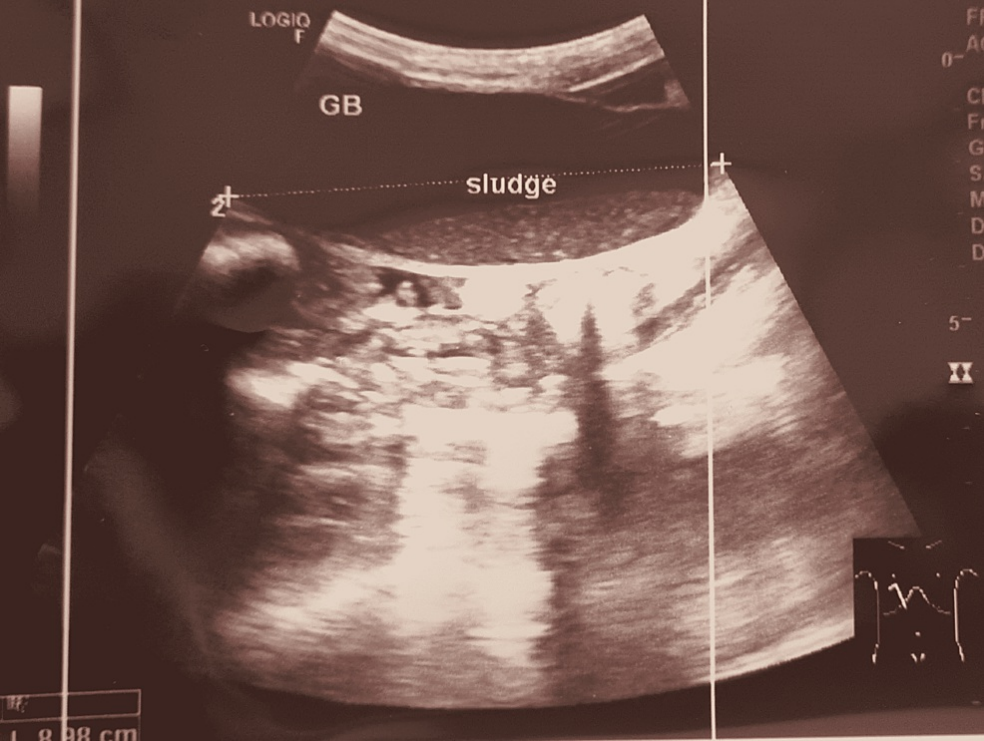
Gallbladder hydrops and bile sludge on abdominal USG. USG: ultrasonography

Blood workup showed normocytic normochromic anemia with hemoglobin of 6.9 g/dL, mean corpuscular volume of 85.4 fL, and mean corpuscular hemoglobin of 30.5 pg, along with increased bilirubin levels (total bilirubin 15.75 mg/dL; direct 14.7 mg/dL, and indirect 1.05 mg/dL) and elevated liver enzymes (serum glutamic-oxaloacetic transaminase 184 U/L and serum glutamic pyruvic transaminase 239 U/L). Further, blood urea nitrogen level was 22 mg/dL, creatinine 0.7 mg/dL, sodium 141 mEq/L, potassium 3.7 mEq/L, and chloride 100 mEq/L (Table 1). Rapid anti-hepatitis C virus and hepatitis B surface antigen tests were negative. Abdominal USG showed a solid mass on the head (caput) of the pancreas, causing dilated intrahepatic and extrahepatic biliary duct (IHBD and EHBD, Figure 1), gallbladder hydrops, and bile sludge (Figure 2), as well as chronic parenchymal disease on the right kidney.

**Table 1:** Laboratory findings.

Characteristics	Result	Units	Normal range
Hemoglobin	6.9	g/dL	13.0–18.0
Leukocyte	8.63	×1,000/µL	4.0–10.0
Platelet	308	×1,000/µL	150–400
Hematocrit	19.3	%	40.0–54.0
Mean corpuscular volume	85.4	fL	81.0–96.0
Mean corpuscular hemoglobin	30.5	pg	27.0–36.0
Mean corpuscular hemoglobin concentration	35.8	g/L	31.0–37.0
Total bilirubin	15.75	mg/dL	0.2–1
Direct bilirubin	14.7	mg/dL	0.1–0.4
Indirect bilirubin	1.05	mg/dL	0.6–0.8
Aspartate transaminase	239	U/L	0–42
Alanine transaminase	184	U/L	0–37
Blood urea nitrogen	22	mg/dL	10.0–50.0
Serum creatinine	0.7	mg/dL	0.3–1.2
Sodium	141	mmol/L	130–145
Potassium	3.7	mmol/L	3.5–5.5
Chloride	100	mmol/L	95–108

**Figure 3:**
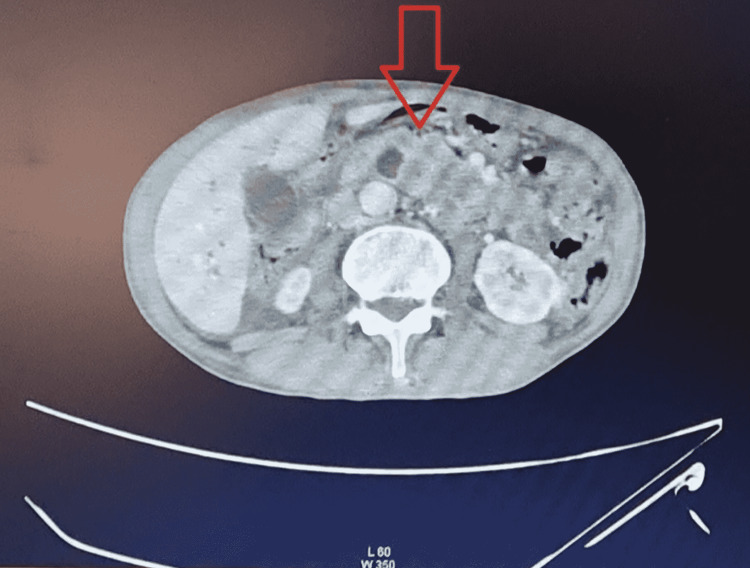
Mass on the pancreatic head in contrast-enhanced abdominal CT scan. CT: computed tomography

Based on the abdominal USG findings, a contrast-enhanced abdominal CT scan and CA 19-9 tests were performed. The contrast-enhanced abdominal CT scan showed features of dilated IHBD caused by a mass on the head of the pancreas; other abdominal organs were within the normal limit (Figure 3). CA 19-9 level was relatively low, 2.06 U/mL. Open biopsy of the pancreas, CA 125, and sialylated Lewis antigen (Sla) were not performed due to the limitation of the hospital’s facility. Finally, the patient was diagnosed as highly suspicious for caput pancreas tumor because the diagnosis was unclear due to the limitations of our facilities. Subsequently, he was referred to Rumah Sakit Umum Pusat (RSUP) Sanglah for further diagnostic measures and treatment.

## Discussion

Generally, CA 19-9 level is increased in pancreatic cancer. In normal pancreatic tissue, protein glycosylation produces end-products, which function as a lubricant for pancreatic ducts. However, an aberrant process occurs in cancer tissues, leading to the production of CA 19-9 [6]. Currently, CA 19-9 or Sla are the only tumor markers approved by the Food and Drug Administration (FDA) in the United States to support the diagnosis of pancreatic cancer [2]. We did not check Sla levels because this tumor marker is not available yet in Indonesia, especially in Bali.

In addition to diagnostic measures, CA 19-9 levels can also be used to determine the prognosis of pancreatic cancer. CA 19-9 levels are inversely related to the survival rate of patients with pancreatic cancer [2,7], with an optimal threshold of ≥338,45 U/mL [8]. Other studies found that a higher threshold of CA 19-9 level was correlated with the survival rate (≥ 800 U/mL) [9]. Based on the correlation, CA 19-9 levels can be used for treatment monitoring and can be combined with clinical and radiological findings to monitor response to therapy and recurrence [10,11]. CA 19-9 levels can also be used to determine whether the pancreatic tumor is resectable, in which decreased CA 19-9 levels increase the likelihood of tumor resection [12].

However, there are some cases in which CA 19-9 is unreliable in diagnostics, prognosis, and therapeutic monitoring because of the inconsistent levels, as reported previously [9-12]. In this case, the patient was suspected of having pancreatic cancer due to a mass in the epigastric region and gradual non-specific symptoms over the last year. There were also symptoms and signs of biliary system obstruction as the patient experienced jaundice, changes in urine and stool, hyperbilirubinemia, and was found to have IHBD and EHBD dilatation on abdominal USG. Based on the imaging findings, the cause of obstruction was a tumor of the head of the pancreas. However, the CA 19-9 levels were relatively low (2.06 U/mL), which was inconsistent with the typical findings of pancreatic cancer.

This phenomenon may occur in populations with Lewis^⍺-β-^ genotype. These populations are not able to express Lewis antigen and CA 19-9; therefore, CA 19-9 investigation for diagnostic and therapeutic measures cannot be reliably used. As much as 10% of the global population has a Lewis^⍺-β-^ genotype, consisting of approximately 5-10% Caucasians and 22% non-Caucasians [3,13,14]. In such populations, there is a genetic deficiency to produce fucosyltransferase enzyme; therefore, CA 19-9 cannot be produced [15]. Epidemiological studies regarding the percentage of the negative Lewis population in Indonesia are yet to be conducted.

Some contradictory findings have shown that not all negative Lewis antigen patients are non-secretors of CA 19-9; therefore, CA 19-9 can be utilized in these populations [16]. In negative Lewis antigen populations, CA 19-9 has a sensitivity of 48.6% and specificity of 95.5% when using a threshold that is much lower than the threshold for positive Lewis populations (1.8 U/mL) [17]. Further studies are needed regarding the utilization of CA 19-9 in the negative Lewis populations that can secrete CA 19-9.

There are prognostic implications for most pancreatic cancer patients with relatively low CA 19-9 levels. Isacoff et al. found that a subgroup of pancreatic cancer patients (n = 13) with normal CA 19-9 levels (≤36 U/mL) at the start of chemotherapy had a longer mean survival rate (30.5 months) compared to most cases with increased CA 19-9 (n = 52; 17 months) [18]. However, this study was conducted in only one institution, and most patients sampled in the study had received previous therapy, either chemotherapy or tumor resection. Therefore, low CA 19-9 levels might be caused by previous treatment.

A study by Luo et al. [19,20] also reported a similar result to Isacoff et al. [18], in which pancreatic cancer patients with normal CA 19-9 levels had a higher survival rate compared to cancer patients with increased CA 19-9 levels, regardless of the cancer stage. Interestingly, pancreatic cancer patients staged III-IV with normal CA 19-9 levels had a higher five-year survival rate (15.4%) compared to stage I-II with increased CA 19-9 levels (13.8%) [19]. Although the prognosis is better and less aggressive compared to pancreatic cancer with increased CA 19-9 levels, pancreatic cancers with normal CA 19-9 levels are less responsive to gemcitabine-based chemotherapy in stage III-IV cancers [19]. Despite the large sample size, this study was a retrospective study; therefore, the findings need to be confirmed with randomized controlled trials.

The low CA 19-9 levels associated with better prognosis might be explained by a hypothesis that CA 19-9 supports any cancer cell metastasis by binding to E-selectin, an adhesion receptor found on the surface of endothelial cells. In addition, according to another hypothesis, CA 19-9 specifically supports the metastasis of pancreatic cancer cells [19,20]. A study by Liu et al. reported contraindicating findings. Overall, 11.7% of 853 patients involved in the study had the negative Lewis genotype with an average survival rate of 7.4 months, significantly different from positive Lewis patients (13.3 months, p < 0.001). Furthermore, there was a higher rate of metastasis in negative Lewis patients (p = 0.004). The mean CA 19-9 levels in negative Lewis patients was 106.0 ± 273.1 U/mL, significantly lower than positive Lewis patients (499.7 ± 635 U/mL, p < 0.001) [21].

An interesting finding of the study was that the CA 125 levels in negative Lewis patients were higher (251.9 ± 642 U/mL) than in positive Lewis patients (135.8 ± 401.6 U/mL, p < 0.001) [21]. Therefore, other tumor markers may be used in cases of low CA 19-9. Although more often used in ovarian cancer, CA 125 is shown to have significant clinical implications in pancreatic cancer. CA 125 is known to be superior in determining whether pancreatic cancer is resectable compared to CA 19-9. CA 125 also correlates with the metastasis-related burden [22].

CA 125, also known as mucin 16 (MUC16), has been recommended by the Chinese Study Group for Pancreatic Cancer for diagnosis, especially in conditions where the CA 19-9 levels are negative and can be used as an additional workup for CA 19-9, especially in hyperbilirubinemia conditions [23]. CA 125 has been shown to be related to metastatic pancreatic cancer with a threshold of 18.4 U/mL [24]. CEA, another tumor marker, can predict the prognosis of advanced pancreatic cancer at diagnosis with a threshold of 7.0 ng/mL [25]. CA 242 is also increased in pancreatic cancer and other digestive cancers; however, it can only be used to monitor response to therapy in advanced cancer and monitor recurrence. However, CA 242 is not effective in detecting early-stage cancer [26].

A combination of several tumor markers may increase the sensitivity and specificity. Combined CA 125 and CA 19-9 increases specificity and sensitivity for pancreatic cancer that has not been previously diagnosed [2]. Although many tumor markers and combinations have been proposed by various studies, none of them can be used in clinical practice other than CA 19-9 because they have not been standardized [2].

## Conclusions

In general, CA 19-9 levels are elevated in pancreatic cancer and have diagnostic, prognostic, therapeutic, and recurrence monitoring benefits. However, there are some cases of pancreatic cancer in which the CA 19-9 test is not reliable, as reported in this case. This phenomenon occurs in negative Lewis antigen populations, with contradicting prognostic implications. In such cases, alternative tumor markers such as CA 125 and CEA or a combination of various tumor markers can be used to increase sensitivity and specificity in diagnosing pancreatic cancer.
